# Global Pollution of Surface Waters from Point and Nonpoint Sources of Nitrogen

**DOI:** 10.1100/tsw.2001.326

**Published:** 2001-11-06

**Authors:** G. van Drecht, A.F. Bouwman, J.M. Knoop, C. Meinardi, A. Beusen

**Affiliations:** National Institute of Public Health and the Environmrnt, Bilthoven, The Netherlands

**Keywords:** ammonia, aquifer, denitrification, groundwater, leaching, nitrate, nitrogen, runoff, surface water

## Abstract

Global 0.5- by 0.5-degree resolution estimates are presented on the fate of nitrogen (N) stemming from point and nonpoint sources, including plant uptake, denitrification, leaching from the rooting zone, rapid flow through shallow groundwater, and slow flow through deep groundwater to riverine systems. Historical N inputs are used to describe the N flows in groundwater. For nonpoint N sources (agricultural and natural ecosystems), calculations are based on local hydrology, climate, geology, soils, climate and land use combined with data for 1995 on crop production, N inputs from N fertilizers and animal manure, and estimates for ammonia emissions, biological N fixation, and N deposition. For point sources, our estimates are based on population densities and human N emissions, sanitation, and treatment. The results provide a first insight into the magnitude of the N losses from soil-plant systems and point sources in various parts of the world, and the fate of N during transport in atmosphere, groundwater, and surface water. The contribution to the river N load by anthropogenic N pollution is dominant in many river basins in Europe, Asia, and North Africa. Our model results explain much of the variation in measured N export from different world river basins.

### INTRODUCTION

Anthropogenic activities have markedly altered the Earth's nitrogen (N) cycle by increasing the natural rate of N fixation compared to preindustrial times[1]. N fixation is the transformation of the highly abundant but biologically unavailable form dinitrogen ($N_2$) in the atmosphere to "reactive" N forms. Biological N fixation occurs by specialized bacteria and algae, which can be either free-living or in symbiotic relationships with higher plants, especially legumes. Further N fixation processes are lightning and N fertilizer and energy production. Depending on the fixation process, $N_2$ is converted to reduced (biological N fixation and industry, mainly N fertilizer production) or oxidized (lightning and energy production) N forms. Part of the fixed reduced N is oxidized in industry to produce nitric acid ($HNO_3$). In the biosphere and atmosphere various reduction and oxidation processes occur, and the valence of N may range from –3 to +5 in many different chemical forms: ammonia ($NH_3$) and ammonium ($NH_4^+$), nitrite ($NO_2^-$), nitrate ($NO_3^-$), nitrogen oxides (NO and $NO_2$), $N_2O_5$, and nitrous oxide ($N_2O$). During denitrification most $NO_3^-$ is reverted back to $N_2$.

Prior to human intervention, the primary N fixation processes were biological N fixation, accounting for around 195 Tg N year$^{-1}$[2] (Tg = teragram; 1 Tg = $10^{12}$ g) in terrestrial ecosystems and 30 to 300 Tg N year$^{-1}$ in marine systems, and chemical fixation by lightning accounting for perhaps 10 Tg N year$^{-1}$[1,3]. The human-induced input of reactive N into the global biosphere may be ~150 Tg N year$^{-1}$ (80 Tg N year$^{-1}$ during N fertilizer production; 45 Tg year$^{-1}$ by biological N fixation; ~30 Tg N year$^{-1}$ during energy production).

Human activities have also increased the mobility of reactive N within and between terrestrial and aquatic ecosystems and the atmosphere[3]. While N pollution occurs at the local level, its influence spreads regionally and globally as it moves through water and air across political and geographic boundaries.

The fate of the anthropogenic reactive N is uncertain, since only about half of it can be accounted for; the other half is either retained on continents in groundwater, soils, or vegetation, or denitrified to $N_2$[1].

N pollution of surface waters has been increasing during the last decades[4,5]. High levels of $NO_3^-$ in drinking water are a cause for concern in some industrialized regions such as Western Europe. This led the European Union to adopt standards for acceptable concentrations in water intended for human consumption. In the EC Directive on Drinking Water 80/778, the maximum admissible concentration of $NO_3^-$ is 50 mg l$^{-1}$, with a "guide level" of 25 mg l$^{-1}$. $NO_3^-$ concentrations in surface waters elsewhere in the world may also be approaching high levels[4].

In this paper, we investigate global N pollution of surface waters and the fate of this N for the situation in the mid 1990s. We consider total N, i.e., $NO_3^-$, $NO_2^-$, $NH_4^+$, and dissolved and particulate organic N. N in surface waters stems from point sources and nonpoint sources. Point sources are primarily associated with human sewage and industrial sources, generally located in urban areas. Nonpoint N pollution of surface waters comprises all N that enters the water in a diffuse manner and is associated with the N use in agricultural land and the disturbance of natural ecosystems primarily by atmospheric N deposition.

In recent studies simple empirical (regression) models were employed relating measured $NO_3^-$ discharge from a number of rivers to population density (point sources) and N loads from atmospheric N deposition and N fertilization[6,7].

We developed a model that contains hydrologic and hydrodynamic characteristics and chemical and biological transformations to describe the various pathways of N loss and transport of total N from point and nonpoint sources to groundwater, rivers, and the river mouths. The data and methodology used for calculating the various N flows are discussed in following section, and the results and conclusions are presented in subsequent sections.

### METHODS AND DATA USED

The procedure for calculating riverine N discharge is presented in Fig. 1. The calculations of the N pollution for point and nonpoint sources are discussed in the next two sections. Further sections subsequently discuss denitrification in the rooting zone and nitrate leaching, along with delay and denitrification loss during groundwater transport and riverine N transport and instream N transformations. The global data with 0.5- by 0.5-degree resolution used in this study are presented in Table 1.

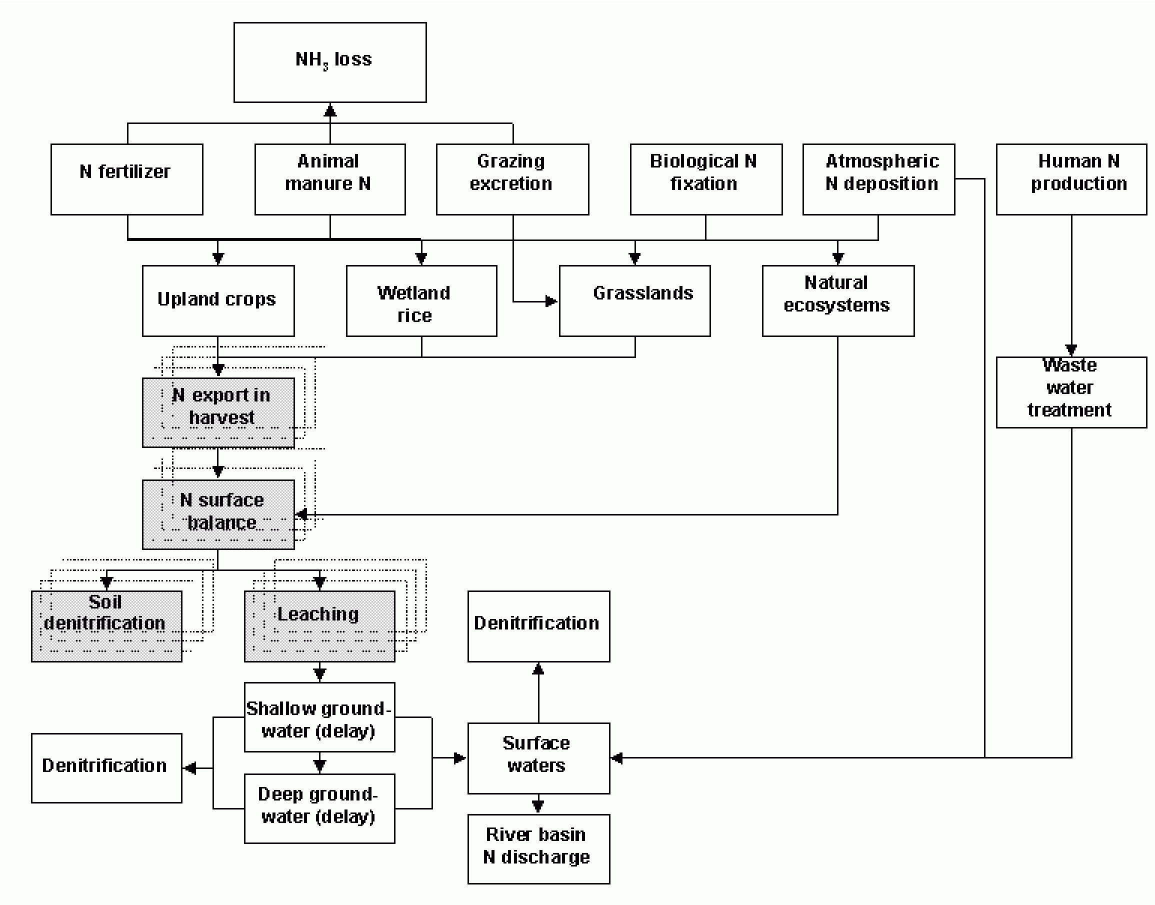

**FIGURE 1.** Calculation of riverine N discharge from point and nonpoint sources of N.

**TABLE 1**
**Global 0.5- by 0.5-Degree Resolution Databases Used**

| Type | Source |
|---|---|
| Runoff, river basins | Various data sets[42]; runoff was partitioned as described elsewhere[43]. |
| Climate | Global climate database[44] using assumed minimum annual precipitation of 1 mm and minimum runoff of 5% of precipitation. |
| Geological formations | Global map based on various sources[44]. |
| Soil characteristics | Total available soil water holding capacity, soil texture, organic carbon, pH, CEC, and drainage from the WISE database[45]. |
| Population density | Densities[46] combined with national urban and rural population data[47]. |
| Land use | Area use of grassland, wetland rice, upland crops, and natural ecosystems[a] per grid cell[48] updated with data for 1995[13]. Fertilized grasslands were allocated to (fractions of) grassland areas using country data on fertilized areas[12]. |
| Atmospheric N deposition | Simulated N deposition[17], converted from the original model resolution of 5 by 5 degrees to 0.5- by 0.5-degree grids using a smoothing filter; we assumed that 50% of $NH_3$ emissions are redeposited within 0.5 degree grid cells[19] to include short-range dry-deposition of $NH_3$. |

[a] Large areas of natural ecosystems are influenced by atmospheric N deposition, and other parts are managed. However, for simplicity we refer to all nonagricultural systems as natural.

### Point Sources of N

The human daily N emission ranges between 8 and 19 g N per capita[8] including N from households and industrial wastes. Because we include industrial emissions, and food consumption is generally above the minimal obligatory level, the lower limit of the range exceeds the "maintenance" level of 4 to 5 g day$^{-1}$ capita$^{-1}$[9]. Based on these extremes, the emission rate for each country is calculated as a function of per capita gross domestic product (GDP) in 1995:

$$N_{emission} = 8 + 11 \cdot \left( \frac{GDP}{46000} \right)^{0.5} \quad (1)$$

The world's highest value is for Switzerland with a GDP of U.S.$46,000. For each country we used data on GDP per capita[10], the sanitation coverage for urban and rural populations, and the fraction of sewage N discharged to surface waters and that removed by water treatment from a recent inventory[11]. The sewage N that is not discharged to surface waters (for example, human waste in latrines, cesspools, and chemical toilets) was assumed not to reach the groundwater or surface waters. We recognize that this may cause an underestimation of the impact of the point sources, particularly in rural areas.

### Nonpoint Sources of N

The steady-state annual mean surface balance for N is the difference between the sum of N inputs (N fertilizer and animal excreta, N fixation, and atmospheric N deposition) and N outputs ($NH_3$ volatilization and removal of N from the field by crop harvesting or grazing). The basis of the calculations for nonpoint sources is the distribution of wetland rice, upland crops, grasslands, and natural ecosystems (Table 1).

#### *Use of N Fertilizers*

We used country data on fertilizer N application rates for crops and grasslands[12] and total N fertilizer use[13]. N application rates were allocated to grid cells with areas of wetland rice, upland crops, or fertilized grassland.

#### *Management of Animal Manure*

The N excretion by cattle, pigs, poultry, sheep, and goats was calculated according to a recent inventory[14] using country animal population data for cattle, pigs, poultry, sheep, and goats[13]. We used estimates for world regions for the fraction of N excreted during grazing in pastures and the fraction of stored or collected animal manure[14]. For some countries these estimates were modified on the basis of an inventory of world livestock production systems[15]. Part of the stored or collected manure is used as a fuel or for other purposes, and the remainder is available for application to grasslands and crops. For most developed countries, we assumed that 50% of the available animal manure is applied to crops[16]. Using this estimate caused a negative N balance in many developing countries; therefore, we assumed that in most developing countries 95% of the available manure is applied to crops, thus accounting for stubble grazing in croplands and the lower importance of grass vis-à-vis the crops in developing countries[15]. Numerous exceptions had to be made to this rule based on the N balances. We assumed that application rates are equal for all crops. Application of animal manure to grasslands (50% of available manure in developed and 5% in developing countries) and cattle grazing is assumed to take place primarily in mixed farming systems, occurring in nonmarginal grasslands in grid cells with a crop coverage of >15% (developing countries) to >35% (developed countries). For a number of countries where grassland-based livestock production systems are dominant[15], we considered all grid cells with nonmarginal grass coverage, irrespective of the crop coverage. Sheep and goats are assumed to graze in all grid cells with grass coverage, including marginal grasslands.

### Biological N Fixation

Biological fixation of atmospheric N by leguminous crops was calculated from the N content of the harvested products for pulses and soybeans (from crop production data, dry matter content, and N content). We assumed that total biomass of pulses and soybeans is twice the harvested product, and that an additional amount of N is fixed that is released during the growing season. To obtain the total of N fixation during the growing season we therefore multiplied the N in the harvested product by a factor of 3. For nonleguminous crops we assumed a rate of biological N fixation of 5 kg $ha^{-1}$ $year^{-1}$. Inputs from crop residues were ignored, since this is assumed to be an internal cycle. Biological N fixation in natural ecosystems and grasslands was estimated on the basis of N fixation rates taken from a global inventory[2] in combination with the spatial distribution of natural ecosystems (Table 1).

#### *Atmospheric N Deposition*

The atmospheric N deposition rates for the mid 1990s are from a global chemistry-transport model[17]. This model describes long-range transport and deposition. To include short-range dry deposition of $NH_3$, it was assumed that 50% of $NH_3$ emissions[18] are redeposited within 0.5-degree grid cells[19]. Atmospheric deposition fields cover all agricultural and natural ecosystems. The mean annual N deposition rate for the global land area is 4.5 kg $ha^{-1}$, but there is strong variability, with highest rates occurring in South and East Asia.

#### *Ammonia Volatilization.*

Local 0.5- by 0.5-degree $NH_3$ volatilization rates from applied animal manure and N fertilizers were calculated with a model[20] based on (1) factors related to agricultural management including crop type (i.e., upland crops, wetland rice, and grass), fertilizer type[21], and fertilizer application technique (broadcasting, incorporation, injection, solution); and (2) factors related to environmental conditions (climate, soil pH, and CEC). $NH_3$ volatilization from grazing systems was calculated according to a recent global inventory[18].

#### *N Removal in Harvested Products*

The removal of N from agricultural fields in the harvested product was calculated from the crop production data[13] and crop dry matter and N content[22]. Because of lack of data, we ignored the export of crop residues used as fuel and animal feed. We recognize that in many parts of the world this may lead to a slight underestimation of the N removal from arable fields.

For grasslands, data on production and consumption (by grazing animals or harvesting) are not available. The N use efficiency of crops is 40 to 50% of the N input from fertilizers[23]. For grasslands we assumed a consumption of 60% of the N inputs from animal manure, N fertilizers, biological N fixation, and atmospheric N deposition, minus the $NH_3$ emission. This means that the N consumption as a percent of the N fertilizer and manure inputs exceeds 60%. This is based on the higher efficiency of N uptake as a result of the longer growing period of grass compared to crops. This assumption results in plausible estimates of the N balance surplus in some European countries.

We neglected N removal in natural ecosystems, assuming that most of these systems are mature with no net accumulation of biomass. This would be incorrect for many managed, seminatural, or disturbed natural ecosystems where net uptake of N occurs. However, we consider this error acceptable considering the low N inputs compared to agricultural systems.

### Denitrification in the Rooting Zone and Nitrate Leaching

Inputs of all reduced N compounds were assumed to be nitrified to $NO_3^-$ in soils. Hence, the quantity of soil $NO_3^-$ equals the long-term annual average surface balance surplus. $NO_3^-$ is subject to denitrification and, since it is highly mobile in soils, it is leached during periods with excess precipitation (precipitation > evapotranspiration). We estimated the denitrification loss using an approach which combines the effect of temperature, soil conditions, and soil hydrological conditions on denitrification and leaching.

Mineralization and denitrification are favored by high temperatures, while these processes slow or even stop at low temperatures. This temperature effect ($f_T$) is calculated according to the Arrhenius equation after Shaffer et el.[24]:

$$f_T = 1.68 \cdot 10^9 \ exp\left(\frac{-e_a}{R \cdot T}\right) \tag{2}$$

where $e_a$ is the activation energy ($54.4284 \times 10^3$ J $mol^{-1}$), $T$ is the mean annual temperature (K), and $R$ is the molar gas constant (8.3144 J $mol^{-1}$ $K^{-1}$). The annual mean number of moisture volumes ($mv$) that percolate through the soil is used as a proxy for leaching, calculated according to:

$$mv = \frac{pe}{tawc} \tag{3}$$

where $pe$ is the precipitation excess (m $year^{-1}$) and $tawc$ is the total available water capacity (m). The combined effect of temperature and precipitation excess ($f_{T,mv}$) is calculated as:

$$f_{T,mv} = \frac{f_T}{mv} \tag{4}$$

In dry climates the water percolation through the soil is slower and the residence time of soil $NO_3^-$ longer than in humid climates. The formulation of $f_T$ implies that N transformation rates are higher in tropical than in temperate climates. In arid climates percolation rates are low ($pe < tawc$; $mv < 1$) and $f_{T,mv} > f_T$ , so $NO_3^-$ leaching is limited. In humid climates leaching rates are high ($pe > tawc$; $mv > 1$) and $f_{T,mv} < f_T$, so leaching rates are high.

Denitrification rates also depend on soil water and oxygen status. Fine-textured soils have more capillary pores and hold water more tightly than do sandy soils. As a result, anaerobic conditions favoring denitrification may be more easily reached and maintained for longer periods within aggregates of fine-textured than in coarse-textured soils. Soil drainage conditions also influence soil aeration. Finally, soil oxygen is consumed by root respiration and microbial activity. Oxygen consumption by microorganisms is driven by the supply of carbon and water availability. Here we use soil organic carbon content as a proxy for the carbon supply. The effect of soil texture ($f_{texture}$), soil organic carbon content ($f_{soc}$) and drainage ($f_{drain}$) on soil moisture and oxygen conditions was included as follows:

$$f_{den} = f_{T/mv} + f_{texture} + f_{drain} + f_{soc} \tag{5}$$

Since denitrification can not exceed the surplus of the surface N balance, the maximum value of $f_{den}$ was assumed to be 1. The values used for $f_{texture}$, $f_{drain}$, and $f_{soc}$ are given in Table 2.

For wetland rice systems, the effects of soil texture, soil organic carbon, drainage, and precipitation surplus were ignored, because these systems are assumed to be predominantly anaerobic. Since rice is generally produced in subtropical and tropical climates, we assumed that denitrification is always 75% of the surface N balance surplus ($f_{den} = 0.75$). This value was selected on the basis of measurements indicating that about 30% of the total N input is lost by denitrification[25,26,27,28,29,30,31,32].

The leaching of $NO_3^-$ from the rooting zone to the subsoil with the downward water flow is calculated as surface balance N surplus minus denitrification loss:

$$N_{leached} = (1 - f_{den}) \cdot N_{surplus} \tag{6}$$

### Delay and Denitrification Loss During Groundwater Transport

The groundwater flow to draining surface water will generally be a mixture of water with varying residence times in the soil. The $NO_3^-$ concentration of groundwater depends on the period during which the water infiltrated and on the denitrification loss during its transport. Groundwater recharged in historical periods (i.e., >50 years ago, when N use was much lower than it is now) will contribute much less $NO_3^-$ to the surface water than groundwater from more recently infiltrated precipitation with concentrations corresponding to fertilization rates prevalent in the 1990s[33].

The distribution of residence times of the saturated groundwater can be represented as follows[34]:

$$\frac{z}{D} = 1 - e^{-\frac{t}{T_r}} \tag{7}$$

where $z$ is the depth below the top of the aquifer (m), $D$ is the aquifer thickness (m), $t$ is the time since recharge (years), and $T_r$ is the mean residence time (years). $z/D$ represents the fraction of the water delivered by the groundwater during a period of $t$ years. We distinguished between rapid transport of $NO_3^-$ through shallow groundwater to local water courses, and slow transport through deep groundwater towards larger streams. The mean residence time $T_r$ for each layer was calculated as:

$$T_r = \frac{D \cdot p}{ro} \tag{8}$$

where $ro$ is the runoff (in m year$^{-1}$) in each groundwater layer based on the partitioning of $pe$ between shallow and deep groundwater, and $p$ is the effective porosity ($m^3\ m^{-3}$). Values for $p$ and $D$ depend on the characteristics of the aquifer considered. We used values for $p$ as presented in Table 3 and applied these to the geological map of the world.

**TABLE 2**
**Denitrification Fractions for Soil Texture ($f_{texture}$), Soil Organic Carbon ($f_{SOC}$), and Soil Drainage ($f_{drain}$)**

| Soil Texture Class | $f_{texture}$ (–) | Soil Drainage Class | $f_{drain}$ (–) | Soil Organic Carbon Content | $f_{SOC}$ (–) |
|---|---|---|---|---|---|
| Coarse | 0.0 | Excessively–well drained | 0.0 | < 1% | 0 |
| Medium | 0.1 | Poorly drained | 0.3 | 1–3% | 0.1 |
| Fine | 0.2 | Very poorly drained | 0.4 | 3–6% | 0.2 |
| Organic | 0.3 | Organic soils | 0.5 | 3–50% | 0.3 |
| | | | | 6–50% | 0.4 |

**TABLE 3**
**Effective Porosity for Different Aquifer Types**

| Type of Material | Permeability | Effective Porosity ($p$) ($m^3/m^3$) Shallow Aquifers | Deep Aquifers |
|---|---|---|---|
| Unconsolidated sedimentary | Good | 0.30 | 0.35 |
| | Poor | 0.15 | 0.20 |
| Consolidated sedimentary | Good | 0.20 | 0.20 |
| | Good | 0.10 | 0.10 |
| Igneous and metamorphic rock | Medium | 0.05 | 0.05 |
| | Poor | 0.02 | 0.02 |

*Note:* Modified from a European study[37].

Shallow transport is assumed to occur in the upper 5-m layer of the saturated zone. For deep groundwater flow, we use a value for $D$ of 50 m. Unconsolidated aquifers with $D > 100$ m are exceptional, because the depositional processes constituting them commonly occurred during a limited geological time span. Consolidated layers may be much thicker than unconsolidated aquifers. However, these generally do not transmit water over their full depth, because water transport mainly occurs in fractured zones with open fissures. Open fissures are generally not present below depths of 100 m due to the overburden[35]. Therefore, a value of 50 m for $D$ for all aquifers is a reasonable estimate in most cases, and possible errors are acceptable, considering the purpose and scale of this global study.

We assumed that water passes through the shallow to the deep groundwater layer. Hence, the $NO_3^-$ concentration in the top the shallow groundwater layer equals the concentration of the water outflow from the rooting zone, and the concentration in the upper part of the deep groundwater equals that of the outflow from the shallow groundwater.

The concentration after groundwater transport in each layer is calculated by combining the residence time, history of N inputs, and denitrification loss as follows:

$$\frac{C_{out}}{C_{in}} = \frac{1}{1 + T_r \cdot (\alpha + k)} \tag{9}$$

where $C_{in}$ is the concentration of the water inflow (g $m^{-3}$), $C_{out}$ is concentration of the water outflow (g $m^{-3}$), $a$ is the coefficient representing the history of the surface N loading, and the coefficient $k$ represents the denitrification rate. Eq. 9 can be found by integration over $0 < z < D$ of the outflow concentration of a single stream line that has entered the top of the aquifer $t$ years ago with an initial inflow concentration of $C_{in} \times e^{-a.t}$. The coefficient $a$ is calculated as $\ln(2)/dt50_{sl,c}$; the half-life for the history of N inputs, $dt50_{sl,c}$, is based on historical data on N fertilizer use for world regions from fertilizer statistics[13]; $k$ is calculated as $\ln(2)/dt50_{den}$; $dt50_{den}$ is the half-life of groundwater N. The $NO_3^-$ concentration of the outflow from shallow aquifers is reduced by assuming a half-life of 2 years, and for deep groundwater in mostly coarse materials a half-life of 3 years[36,37].

### Riverine N Transport and Instream N Transformations

The total of N sources from sewage, atmospheric deposition to open water, and $NO_3^-$ flows from shallow and deep groundwater are input to the surface water within each grid cell. Once N enters the aquatic system, instream N transformations will occur as a result of both physical and chemical discontinuities in the stream. Instream N transformations are primarily concerned with metabolic processes, which remove N from the stream water by transferring it to the biota, atmosphere, or stream sediments.

Various factors influence N loss and retention, including temperature, availability of organic matter, $NO_3^-$ concentration in stream water overlying stream sediments, and the stream flow regime and water residence time. Catchment-driven models generally apply export coefficients, which are useful for obtaining rough estimates of annual nutrient loads[5]. Here, a global river export coefficient of 0.7 is adopted (implying retention and loss of 30% of the N) based on recent studies[37,38,39].

## RESULTS AND DISCUSSION

The results of our analysis for ten major rivers varying in size, runoff, N load, and the relative importance of point and nonpoint sources of N are presented in Table 4. The Amazon, Zambezi, and Zaire are characterized by large areas of natural ecosystems, a small population, and relatively low level of agricultural activity. The Nile drains a large area, but population and agricultural activity are more important and are concentrated in only a fraction of the total area. The contribution of sewage N at the mouth of the river Nile is, therefore, rather high. For the other rivers, population and agricultural activity play a more important role than do natural ecosystems.

There are important differences in the fraction of the N inputs that is lost in soils and during transport through groundwater and surface waters. This loss exceeds 90% in the Amazon and Mississippi river basins, while for the other basins it ranges between 77 and 86%. This variation is mainly caused by differences in geology, climate and soil conditions, and sanitation coverage and water treatment within the river basins.

**TABLE 4**
**Drained Land Area, Runoff, Population, N Inputs (in Natural and Agricultural Systems, from Sewage and Atmospheric N Deposition) and Calculated Export of N at River Mouth from Natural and Agricultural Systems and Sewage for Ten Selected Rivers**

| | | | | | Inputs | | | | Export[a] | | | | |
|---|---|---|---|---|---|---|---|---|---|---|---|---|---|
| River | Drained land area ($10^6$ km$^2$) | Runoff (1000 km$^3$ year$^{-1}$) | Population (Inh./km$^2$) | Rural Pop. (%) | Total (kg N km$^{-2}$ year$^{-1}$) | Natural (%) | Agriculture (%) | Sewage (%) | Total (Gg year$^{-1}$) | Total (kg N km$^{-2}$ year$^{-1}$) | Natural (%) | Agriculture (%) | Sewage (%) |
| Mississippi | 3.2 | 0.6 | 21 | 44 | 7489 | 9 | 89 | 1 | 1910 | 597 | 27 | 63 | 10 |
| Amazon | 5.8 | 6.4 | 4 | 68 | 3034 | 83 | 17 | 0 | 4001 | 692 | 93 | 6 | 1 |
| Nile | 3.7 | 0.3 | 38 | 71 | 3601 | 31 | 67 | 2 | 998 | 268 | 43 | 37 | 20 |
| Zaire | 3.6 | 1.2 | 16 | 76 | 3427 | 81 | 18 | 0 | 2290 | 632 | 90 | 9 | 1 |
| Zambezi | 1.9 | 0.4 | 14 | 74 | 3175 | 52 | 47 | 1 | 641 | 330 | 68 | 25 | 6 |
| Rhine | 0.2 | 0.1 | 292 | 10 | 13941 | 15 | 77 | 9 | 459 | 2795 | 21 | 49 | 30 |
| Po | 0.1 | 0.1 | 196 | 29 | 9060 | 11 | 81 | 8 | 185 | 1841 | 17 | 56 | 27 |
| Ganges | 1.6 | 1.2 | 286 | 78 | 9366 | 16 | 81 | 3 | 2051 | 1269 | 30 | 55 | 15 |
| Chang Jiang | 1.8 | 0.9 | 237 | 73 | 11823 | 6 | 92 | 2 | 3959 | 2237 | 9 | 83 | 8 |
| Huang He | 0.9 | 0.1 | 153 | 66 | 5159 | 9 | 88 | 3 | 190 | 214 | 17 | 24 | 59 |

[a] Contributions for each source (natural systems, agriculture, and sewage) were calculated by excluding the other two sources. Atmospheric N deposition has not been calculated separately, because it is difficult to attribute N deposition to its original sources.

The fraction of the N inputs that is lost is also determined by contribution of the different N sources to river N export. Losses during both groundwater and river transport of N stemming from nonpoint sources are much greater than the losses of sewage N, which are transported through river systems only. Therefore, the contribution of sewage to total N export at the river mouth exceeds the fraction of sewage N of the total N inputs in the river basins.

Despite the large losses of N in the groundwater systems, the contribution of agriculture to the N load at the river mouth is dominant in all rivers with high levels of agricultural activity (Mississippi, Ganges, Chang Jiang, Huang He, Rhine, and Po).

We summarized our results for a number of continents and world regions using an N export index that represents the importance of anthropogenic N pollution of rivers relative to N export from areas of "natural" ecosystems (Table 5). It is clear that the world's N export has doubled due to anthropogenic activities (world N export index value of 0.5). The regions where the index value is highest are Europe (0.7; i.e., total export exceeds N export from areas of natural ecosystems by a factor of 3) and

**TABLE 5**
**Land Area, Runoff, Population Density, Total N Inputs and N Export, and the N Export Index[a]**

| Unit | North America | Latin America | Africa | Europe | Former USSR | Asia | Oceania | World |
|---|---|---|---|---|---|---|---|---|
| Land area ($10^6$ km$^2$) | 17 | 21 | 29 | 5 | 24 | 23 | 8 | 127 |
| Runoff (1000 km$^3$ year$^{-1}$) | 5 | 12 | 5 | 2 | 4 | 10 | 1 | 38 |
| Population (Inhabitants km$^{-2}$) | 16 | 21 | 23 | 104 | 15 | 133 | 3 | 42 |
| N inputs (Tg year$^{-1}$) | 49 | 77 | 79 | 34 | 38 | 127 | 17 | 420 |
| N export (Tg year$^{-1}$) | 6 | 11 | 8 | 5 | 3 | 19 | 1 | 54 |
| N export index | 0.5 | 0.4 | 0.4 | 0.7 | 0.5 | 0.7 | 0.4 | 0.5 |

*Note:* We allocated the N export to continents and world regions on the basis of the location of the river mouth. Hence, the area drained may not coincide with the area of the continent or region considered.

[a] Calculated as 1 – R, where R = export of N stemming areas of natural ecosystems/total N export. A value of 0 indicates absence of anthropogenic influence on N export, while values close to 1 indicate that anthropogenic sources >> natural sources. A value of 0.5 means that total N export is twice the export from natural sources.

Asia (0.7). In Africa, Latin America, and Oceania, the N export index is 0.4; hence total N export exceeds N export from areas of natural ecosystems by a factor of 1.6. Within these regions the anthropogenic N export and the index value for river basins varies as a result of characteristic patterns of population density and agricultural activity and intensity. For example, high values were calculated for North Africa where in many river basins much of the population is concentrated close to the river mouths.

We compared our results for the N load at the river mouths with available data[7,40]. The data on water quality from UNEP[40] are from the period January 1, 1994 to December 31, 1996, but the length of the measurement period varies from 1 to 3 years for the different stations. The most common sampling frequency is about 1 month. DIN is the sum of the arithmetic means of concentrations of $NO_3^-$, $NO_2^-$, and $NH_4^+$. $N_{tot}$ is the sum of arithmetic means of $NO_3^-$, $NO_2^-$, $NH_4^+$, and organic N. Caraco and Cole[7] present $NO_3^-$ loads for 35 major rivers, but information on the data and the calculation of river loads is not provided.

The comparison of model results with measurements on DIN concentrations for 18 rivers (Fig. 2a) shows that, with some exceptions, our model explains much of the variation. The comparison of calculated N river load with $NO_3^-$ load[7] in Fig. 2b is difficult, because our modeling results are for total N, including $NH_4^+$ and organic N, but the general trends are in close agreement (Fig. 2b). The comparison also suggests that the export coefficient we used to describe instream N transformations is probably too high. Using a coefficient of 0.4 (or retention of 60%) would yield a better agreement with the measurements (Fig. 2). The only value for total N total (DIN + organic N) for the Narmade River in India (Fig. 2a) shows the importance of the organic N fraction, particularly in countries with no or almost no sewage treatment. In combination with high temperatures, this may cause high denitrification rates leading to significant instream N loss.

The approach described in this paper is fraught with uncertainties and scaling problems in the different modeling steps. With regard to the point sources, we calculated human N emissions with a nonlinear function of per capita GNP. Hence, we ignored variation in incomes within countries and the specific location of industrial activities. Furthermore, data on sanitation coverage and sewage water treatment are uncertain.

There is also considerable uncertainty in the data on the nonpoint sources of N. Data on N excretion and animal waste management systems are based on estimates for world regions, while they may vary between and within countries. Regarding

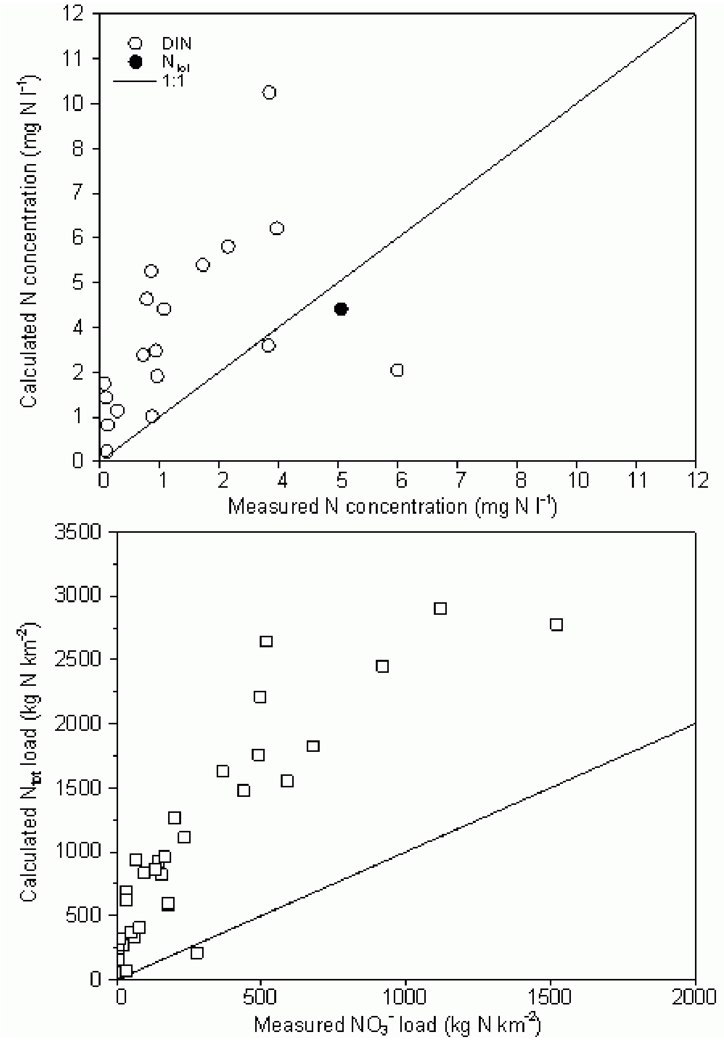

**FIGURE 2.** Comparison of model results with (a) published data on N concentrations[40] and (b) data on $NO_3^-$ load[7] for a number of major world rivers.

the use of N fertilizers and animal manure N, we used country average application rates. Because of sparsity of data we ignored the spatial and temporal heterogeneity in fertilizer management within countries (e.g., differences in N application rates and timing of application between regions and crops) and variation resulting from crop rotations. These problems may cause errors in the calculation of nonlinear soil processes such as ammonia volatilization, denitrification, and leaching. Proper description of these processes at the 0.5- by 0.5-degree resolution is difficult[41]. Finally, atmospheric N deposition and biological N fixation rates are uncertain.

Although it is known that the history of N inputs and age of groundwater strongly determine the $NO_3^-$ content of deep groundwater, there is considerable uncertainty in our estimations of the $NO_3^-$ content of the groundwater discharge to surface waters. Our calculations are based on the assumption of anaerobic conditions in all groundwaters. Perhaps the most uncertain processes in our calculations are the instream N transformations; these are described by one export coefficient for all world rivers, which may not be not appropriate to cover the heterogeneity in conditions controlling N transformations[7].

### CONCLUSIONS

Despite all the uncertainties, our results provide a first insight in the magnitude of the N losses from soil-plant systems and point sources in various parts of the world, and the fate of N during transport in groundwater and surface waters. River basins with risk to eutrophication of rivers, estuaries, and coastal seas are located in all continents, but highest risks are expected in Asia, Europe, and North Africa. In these regions detailed analyses are needed to determine the fate of N on a more local scale.

Relatively simple improvements on our approach can be achieved by (1) estimating the in-stream N transformations on the basis of climatic conditions, the residence time, the size of the river basins, and shape of the riverbeds on the basis of comparison with river export measurements; and (2) improving the estimation of human N emissions, the sanitation coverage, removal of N during sewage water treatment, and the handling of the N that is not directly discharged to surface waters.

| | | |
|---|---|---|
| Received: | July | 23, 2001 |
| Revised: | October | 19, 2001 |
| Accepted: | October | 25, 2001 |
| Published: | November | 6, 2001 |
